# Magnetic‐Field Tunable Intertwined Checkerboard Charge Order and Nematicity in the Surface Layer of Sr_2_RuO_4_

**DOI:** 10.1002/adma.202100593

**Published:** 2021-06-27

**Authors:** Carolina A. Marques, Luke C. Rhodes, Rosalba Fittipaldi, Veronica Granata, Chi Ming Yim, Renato Buzio, Andrea Gerbi, Antonio Vecchione, Andreas W. Rost, Peter Wahl

**Affiliations:** ^1^ School of Physics and Astronomy University of St Andrews North Haugh, St Andrews Fife KY16 9SS UK; ^2^ CNR‐SPIN UOS Salerno Via Giovanni Paolo II 132 Fisciano I‐84084 Italy; ^3^ Dipartimento di Fisica “E. R. Caianiello” Universitá di Salerno Fisciano, Salerno I‐84084 Italy; ^4^ CNR‐SPIN Corso F.M. Perrone 24 Genova 16152 Italy; ^5^ Max‐Planck‐Institute for Solid State Research Heisenbergstr. 1 70569 Stuttgart Germany

**Keywords:** electronic structure, ruthenate perovskites, strongly correlated electron systems, quantum criticality

## Abstract

In strongly correlated electron materials, the electronic, spin, and charge degrees of freedom are closely intertwined. This often leads to the stabilization of emergent orders that are highly sensitive to external physical stimuli promising opportunities for technological applications. In perovskite ruthenates, this sensitivity manifests in dramatic changes of the physical properties with subtle structural details of the RuO_6_ octahedra, stabilizing enigmatic correlated ground states, from a hotly debated superconducting state via electronic nematicity and metamagnetic quantum criticality to ferromagnetism. Here, it is demonstrated that the rotation of the RuO_6_ octahedra in the surface layer of Sr_2_RuO_4_ generates new emergent orders not observed in the bulk material. Through atomic‐scale spectroscopic characterization of the low‐energy electronic states, four van Hove singularities are identified in the vicinity of the Fermi energy. The singularities can be directly linked to intertwined nematic and checkerboard charge order. Tuning of one of these van Hove singularities by magnetic field is demonstrated, suggesting that the surface layer undergoes a Lifshitz transition at a magnetic field of ≈32T. The results establish the surface layer of Sr_2_RuO_4_ as an exciting 2D correlated electron system and highlight the opportunities for engineering the low‐energy electronic states in these systems.

## Graphical abstract

Using ultralow temperature scanning tunneling microscopy and spectroscopy, the low‐energy electronic structure of the surface layer of Sr_2_RuO_4_ is revealed. Small structural distortions lead to radically different properties compared to the bulk with multiple van Hove singularities close to the Fermi energy giving rise to nematic and charge orders. The van Hove singularities can be manipulated by a magnetic field.

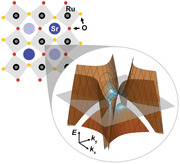

## 1 Introduction

The physical properties of strongly correlated electron materials often vary dramatically with comparatively modest external stimuli,^[^ ^1^ ^]^ evidenced, for example, by magnetic‐field driven metamagnetic transitions,^[^ ^2^, ^3^ ^]^ doping‐induced metal‐to‐insulator transitions^[^ ^4^ ^]^ and superconductivity^[^ ^5^ ^]^ as well as a surprising sensitivity to uniaxial strain.^[^ ^6^ ^]^ The changes in physical properties are usually accompanied by tiny structural distortions often reflecting, or even inducing, the lower symmetry of the new electronic states. This sensitivity of physical properties is exemplified in the perovskite ruthenates. The members of the Ruddlesden–Popper series of strontium ruthenate, Sr_*n*+1_Ru_*n*_O_3*n*+1_, exhibit an exceptional variety of ground states ranging from superconductivity for *n* = 1^[^ ^7^ ^]^ via materials with a rich metamagnetic phase diagram for *n* = 2^[^ ^8^ ^]^ and 3^[^ ^9^ ^]^ to bulk ferromagnetism for *n* → ∞.^[^ ^10^ ^]^ This wide variety of properties is intimately linked to small structural distortions of the RuO_6_ cage. Already in the superconductor Sr_2_RuO_4_, with *n* = 1, a soft phonon mode associated with the octahedral rotation is observed.^[^ ^11^ ^]^ Isoelectronic substitution of Sr by Ca results in a rich phase diagram with metallic and ferromagnetic phases until an antiferromagnetically ordered Mott insulator is reached, where the dominant change to the material is a small rotation and tilting of the RuO_6_ cage.^[^ ^1^, ^4^ ^]^ Understanding the impact of that rotation on the electronic structure therefore provides a key to controlling electronic and physical properties of perovskite ruthenates. A prerequisite to link physical properties to details of the octahedral rotation is an understanding of the low‐energy electronic structure. We here establish the surface layer of Sr_2_RuO_4_ as a clean and well‐controlled 2D model system where this can be achieved and explored.

**Figure 1:**
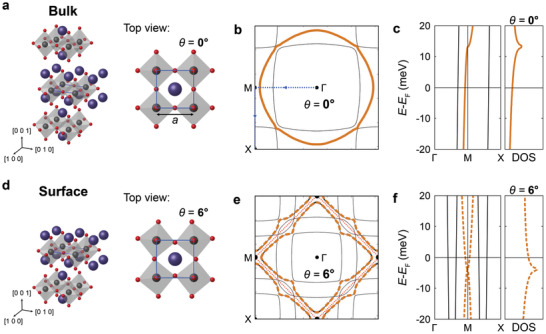
Electronic structure of Sr_2_RuO_4_ with and without octahedral rotation. a) Structural model of the bulk of Sr_2_RuO_4_ (black: Ru, red: O, purple: Sr atoms). Right: top view, with the unit cell of lateral size *a* indicated by a blue square. The octahedra have the same orientation at each lattice site, with an angle of θ = 0°. b) Fermi surface of Sr_2_RuO_4_ with θ = 0°^[^ ^18^ ^]^ (yellow line: d_*xy*_ band, black lines: d_*xz*_/d_*yz*_ bands). c) Electronic structure of bulk Sr_2_RuO_4_. The d_*xy*_ band exhibits a van Hove singularity (vHs) at the M point above *E*_F_, which results in a peak in the density of states (DOS, right). d) Structural model of the surface of Sr_2_RuO_4_ with θ = 6°. The rotation is more apparent in the top view shown on the right and leads to a doubling of the unit cell. The unit cell with θ = 0° is shown as a blue square for comparison. e) Fermi surface with θ = 6° (yellow dashed line: d_*xy*_ band; see Section S4, Supporting Information for details). The surface Brillouin zone is indicated by dotted red lines. f) Electronic structure corresponding to (e). The vHs at the M‐point is pushed below *E*_F_ with the octahedral rotation. The DOS is shown on the right, where the peak occurs below *E*_F_.

The bulk material of Sr_2_RuO_4_ has a tetragonal unit cell with undistorted RuO_6_ octahedra^[^ ^12^, ^13^ ^]^ aligned with the high‐symmetry directions of the crystal (left panel **Figure** **1**a). It is a superconductor with a transition temperature *T*_*c*_ ≈ 1.5K,^[^ ^7^ ^]^ and is known to exhibit strong electron correlations, evidenced by effective masses of the bands between 6 and 17*m*_*e*_ in the normal state.^[^ ^14^ ^]^ Its bulk Fermi surface has been established by quantum oscillations^[^ ^15^ ^]^ and confirmed by angle‐resolved photoemission spectroscopy (ARPES).^[^ ^14^, ^16^, ^17^ ^]^

In Figure 1b, we show its Fermi surface: The d_*xy*_ band (yellow line) has a van Hove singularity (vHs) at the M‐point of the Brillouin zone (BZ) at about 14 meV above *E*_F_ (Figure 1c).^[^ ^19^ ^]^ Upon cleaving, the exposed surface layer exhibits a 6° rotation of the RuO_6_ octahedra^[^ ^20^ ^]^ (Figure 1d). In bulk Sr_2_RuO_4_, a similar octahedral rotation occurs on substitution of Sr by Ca quickly suppressing superconductivity.^[^ ^4^ ^]^ Early STM studies^[^ ^21^, ^22^ ^]^ suggest that the rotation also suppresses superconductivity in the surface layer. The rotation further leads to orbital‐dependent renormalizations close to the Fermi energy.^[^ ^23^, ^24^ ^]^ This rotation doubles the size of the unit cell and leads to a reconstruction of the Fermi surface (Figure 1d), and a shift of the vHs below *E*_F_ (Figure 1e).^[^ ^17^ ^]^ The vHs leads to a peak in the density of states (DOS). In Figure 1c,e) we show schematically the DOS of the unreconstructed (solid line) and reconstructed (dashed line) electronic structure in comparison. The energy of the vHs is seen to be highly sensitive to structural details of the RuO_6_ octahedra. Due to the small interlayer coupling in Sr_2_RuO_4_, the surface reconstruction provides an opportunity to establish the influence of small structural distortions on the electronic structure in a very clean and chemically homogeneous 2D system, and hints at what the leading instabilities of the bulk material are. We here demonstrate that the reconstructed surface of Sr_2_RuO_4_ by itself constitutes a strongly correlated system with its own unique electronic properties: checkerboard charge order, nematicity and four van Hove singularities (vHss) within a few millielectronvolts of the Fermi energy. We can link these phenomena through a phenomenological tight‐binding model based on the bulk electronic structure and incorporating these emergent orders which yields a density of states in excellent qualitative agreement with tunnelling spectra. We demonstrate magnetic‐field tuning of one of the vHss, and extrapolate from our measurements that the surface layer undergoes a Lifshitz transition at 32T.

## 2 Results

### 2.1 Checkerboard Charge Order

**Figure 2:**
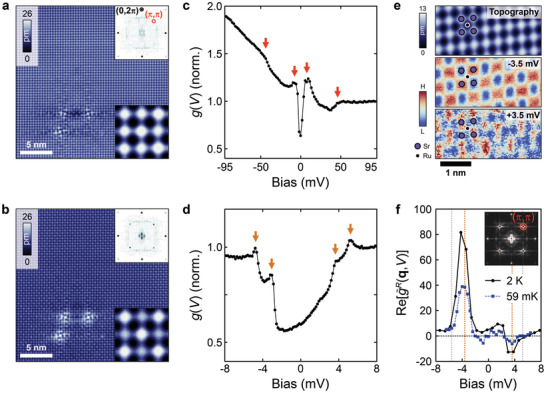
Checkerboard charge order. a) Topography taken at *V* = +5 mV, showing the Sr square lattice (*I*_set_ = 50 pA). Lower inset: enlarged topography. Upper inset: Fourier transformation with Bragg peaks at (0, 2π) (black circle) and (2π, 0). Peaks at (π, π) (red circle) and (−π, π) coincide with the periodicity of the surface reconstruction (reciprocal lattice vectors in units of 1/*a*). b) Topography at *V* = −5 mV, showing a clear checkerboard (*I*_set_ = 50pA), shown in more detail in the lower inset. Upper inset: Fourier transformation showing increase of intensity at (π, π). c) Tunneling spectrum *g*(*V*) measured at *T* = 76 mK (*V*_set_ = 100 mV, *I*_set_ = 265.2 pA, *V*_L_ = 1.75 mV). d) High resolution *g*(*V*) spectrum around *E*_F_ with a gap and four peaks indicated by yellow arrows (*V*_set_ = 8 mV, *I*_set_ = 500.2 pA, *V*_L_ = 155 μV, *T* = 56 mK). e) Top: Topography with a model indicating the positions of the Sr atoms. Bottom: Real‐space *g*(**r**,*V*) maps at *V* = −3.5 mV and *V* = +3.5 mV recorded simultaneously with the topography. At −3.5 mV, a strong checkerboard charge order is observed which has opposite phase at +3.5 mV (*T* = 59 mK, *V*_set_ = 7.0 mV, *I*_set_ = 250 pA, *V*_L_ = 495 μV). f) Energy dependence of the phase‐referenced Fourier transformation ${\tilde{g}}^{R}(q,V)$ at $q_{\text{ckb}}=(\pi ,\pi )$ obtained from maps taken at *T* = 2 K and 59 mK. Two peaks at −3.5 mV and +3.5 mV with opposite phase and a width of ≈1 mV can be seen (see Section S3, Supporting Information for details). Vertical dotted lines indicate the position of the four peaks observed in the average g(r,V) spectrum at 59 mK. Insert: Fourier transformation $\tilde{g}(q,V)$ for the *T* = 2 K map, $q_{\text{ckb}}$ is indicated by a red circle.

**Figure** **2**a shows a topographic image of Sr_2_RuO_4_ at a bias voltage *V* = 5 mV, recorded at a temperature of 76 mK, showing a SrO‐terminated surface^[^ ^25^, ^26^ ^]^ with atomic resolution and demonstrating a low concentration of point defects (less than 0.1%). We find defects at the Ru site with two distinct orientations due to the octahedral rotation in the surface layer. The Fourier transformation of the topography (upper inset in Figure 2a), shows the presence of quasi‐particle interference (QPI), consistent with previous reports,^[^ ^27^ ^]^ as well as Bragg peaks at (0, 2π) and (2π, 0) due to the Sr lattice, and peaks with a low intensity at (π, π) corresponding to the periodicity of the surface reconstruction. When recording topographic STM images at bias voltages of −5 mV, Figure 2b, the appearance changes significantly. The image contrast is now dominated by the presence of a strong modulation of the charge density (lower inset in Figure 2b) which corresponds to a large increase in the intensity of the (π, π) peaks in the Fourier transformation. This modulation has been observed previously at the surface of Sr_2_RuO_4_,^[^ ^25^ ^]^ as well as at that of Sr_3_Ru_2_O_7_.^[^ ^28^ ^]^ However, the octahedral rotation due to the surface reconstruction cannot explain the charge modulation: adjacent Sr and Ru sites are in an equivalent structural environment that can be transformed into each other through a symmetry operation—a mirror operation for Ru and a 90° rotation for Sr. Detailed studies of the surface structure by low‐energy electron diffraction (LEED) do not reveal any additional reconstruction of the surface layer apart from the octahedral rotation,^[^ ^29^ ^]^ leaving the origin of the checkerboard charge order as an open question. We note that the checkerboard charge order is most prominent for small bias voltages, suggesting an electronic origin (see Section S2, Supporting Information). A typical differential conductance spectrum *g*(*V*) in the range ±95 mV is presented in Figure 2c. Here, kink‐ and gap‐like features are observed at ±40 meV and ±5 meV, respectively. The latter is associated with a reduction of the differential conductance by almost 30% in relation to the value at 95 mV. The general shape of the spectrum, as well as the absence of a superconducting gap, is in agreement with previous reports.^[^ ^21^, ^22^, ^27^ ^]^ However, from high‐resolution spectra acquired at temperatures below 100 mK, we find that this gap‐like feature actually exhibits four well defined peaks in the differential conductance within ±5 meV of the Fermi energy (Figure  2d).

The additional modulation of the charge density leads to pronounced signatures in spectroscopic maps: while topographic images obtained at positive bias voltage show predominantly the Sr lattice (top panel in Figure 2e), differential conductance maps exhibit a clear checkerboard charge modulation superimposed to this lattice at bias voltages close to *E*_F_ (second and third panel in Figure 2e). The checkerboard exhibits a contrast inversion across the Fermi energy. We plot in Figure 2f the energy dependence of the intensity of the checkerboard analyzed through a phase‐referenced Fourier transformation (PR‐FT, see Section S3C, Supporting Information), which provides information about the amplitude and phase of the modulation. The amplitude of the checkerboard exhibits a pronounced maximum at −3.5 mV with a width of only about 1mV and a weaker maximum at +3.5 mV of similar width but with opposite phase. Both maxima occur at the same energy as the energy of the inner‐most peaks in the tunneling spectrum (yellow dotted lines in Figure 2f). The phase change is consistent with what one would expect for a charge density wave.^[^ ^30^ ^]^

### 2.2 Nematicity

**Figure 3:**
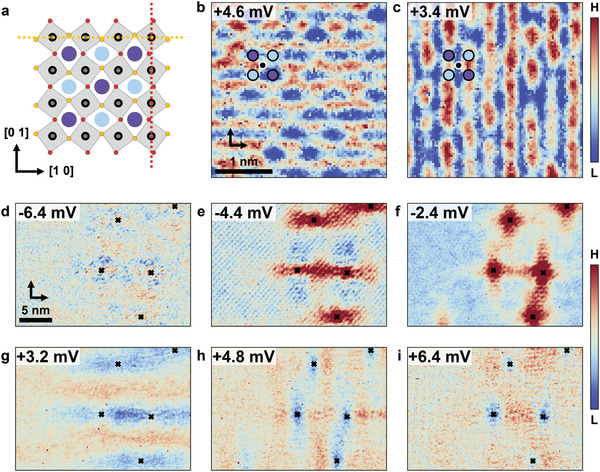
Nematicity and the equivalence of checkerboard charge order with *C*_4_ symmetry breaking. a) Model of the surface atomic structure with the checkerboard charge order on the Sr atoms (purple and light blue circles). The charge order on the Sr lattice combined with the octahedral rotations leads to a broken *C*_4_ symmetry, due to which oxygen atoms along the [10] and [01] directions are in an inequivalent environment. This results in inequivalent hopping amplitudes along the horizontal (yellow) and vertical (red) dashed lines. The color of the oxygen atoms represents whether they are closer to a light blue Sr atom (yellow) or a purple Sr atom (red). b,c) Nematicity in the atomic scale charge modulations (*T* = 1.8 K, *V*_set_ = 7.8 mV, *I*_set_ = 500 pA, *V*_L_ = 370 μV). d–i) Real‐space images showing directional quasi‐particle interference near defects on a longer length scale, the defects are marked by black crosses (*T* = 56 mK, *V*_set_ = 6.4 mV, *I*_set_ = 225 pA, *V*_L_ = 398 μV).

The Sr‐centred checkerboard charge order and nematicity are intimately linked in the surface layer through the octahedral rotation. The octahedral rotation itself preserves *C*_4_ symmetry and does not give rise to an additional charge modulation or nematicity. However, the occurrence of a Sr‐centred checkerboard, as we observe experimentally, and nematicity are equivalent: due to the checkerboard charge order centred at the Sr sites and the octahedral rotation, the oxygen atoms in the Ru plane become inequivalent between the horizontal, [10], and vertical, [01], directions. As shown in **Figure** **3**a, the rotation means that the oxygen atoms connecting Ru atoms in the [01] (vertical) direction (colored in red) are closer to Sr atoms with a decreased charge density (shown dark), whereas oxygen atoms in the [10] (horizontal) direction (shown orange) are closer to Sr atoms with increased charge density. This leads to different hopping amplitudes across those oxygen atoms, indicated in Figure 3a by the yellow and red dashed lines, resulting in nematicity of the electronic states and a reduction from *C*_4_ symmetry to *C*_2_ symmetry (Figure 3a). The converse holds true as well, nematicity on the oxygen sites (indicated by different coloured oxygen atoms in Figure 3a) results in their inequivalence and hence checkerboard charge order on the Sr sites – highlighting the equivalence of the two orders. Therefore, while the octahedral rotation itself gives rise to neither nematicity nor checkerboard charge order, the two become intimately related through the octahedral rotation—so if one occurs, the other will too. This emergent nematicity is confirmed experimentally through unidirectional modulations with atomic periodicity (Figure 3b,c) and anisotropy of the low‐q quasi‐particle interference (Figure 3d–i). The atomic scale symmetry breaking reveals that for changes in the bias voltage *V* by about 1 mV the atomic‐scale unidirectional periodicity changes direction between the high‐symmetry directions [10] and [01] implying a small characteristic energy scale of the nematicity. This is also confirmed in the intensity of the atomic peaks (Figure S5, Supporting Information). The symmetry breaking of long‐wavelength quasi‐particle interference is shown in a real‐space map around four defects in Figure 3d–i (defects marked by black crosses) and reveals a change from predominant quasiparticle scattering along [10] to scattering along [01] and back as a function of energy.

We note that neither nematicity nor checkerboard charge order are captured by the surface structure determined by *I*(*V*)‐LEED.^[^ ^29^ ^]^ Nor does *I*(*V*)‐LEED or our STM data provide any evidence for an orthorhombicity of the surface layer which could give rise to a lowered symmetry.

### 2.3 Tight‐Binding Model for Surface Electronic Structure

**Figure 4:**
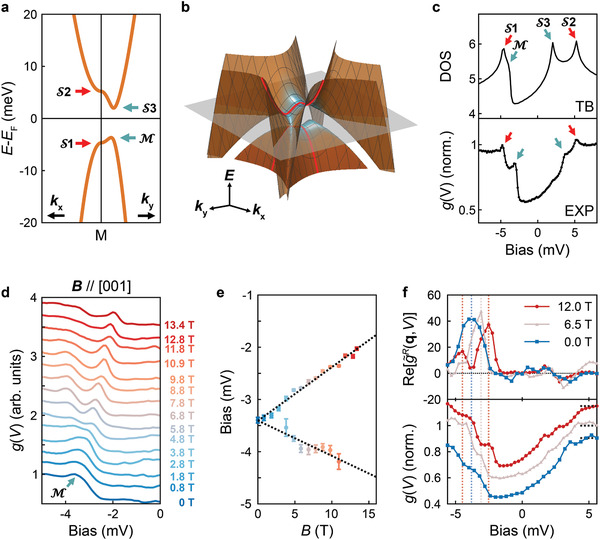
Tight‐binding model and magnetic‐field tuning of van Hove singularities. a,b) Band structure around M from the tight‐binding model including an intraband hybridization potential and nematic order parameter. b) 3D band dispersion around M within 20 meV of *E*_F_. Blue shaded regions indicate the locations of the vHs (labelled S1, S2, S3, and M in (a)). Red lines: path shown in (a), the plane corresponds to *E*_F_. c) Upper panel: Density of states from the model, with four vHss. Bottom: tunneling spectrum of Figure 2d with gap‐like structure and four distinct peaks associated with the vHss. d) Tunneling spectra (*T* = 76 mK) in magnetic fields (*B*||*c*) from 0T to 13.4T (*V*_set_ = 5 mV, *I*_set_ = 225 pA, *V*_L_ = 100 μV). e) Peak position of M as a function of field extracted from fits (see Section S5, Supporting Information) revealing splitting and linear magnetic field dependence with g* ≈ 3 (error bars: 95% confidence intervals). f) Top: energy dependence of the Fourier peak at in the PR‐FT at *B* = 0T, 6.5T and 12T (see Section S3C, Supporting Information for details). Bottom: spatially averaged differential conductance spectra *g*(*V*). The vertical dashed lines area guide to the eye, showing correspondence of features in *g*(*V*) and the PR‐FT. (Spectra are normalized and vertically shifted for clarity, the short dashed gray lines indicate *g*(*V*) = 1). (*T* = 76 mK, *V*_set_ = 5.6 mV, *I*_set_ = 225.2 pA, *V*_L_ = 300 μV map at 6.5T; *T* = 500 mK, *V*_set_ = 5.6 mV, *I*_set_ = 200.5 pA, *V*_L_ = 280 μV for map at 12 T).

To understand the microscopic consequences nematicity and the observed charge modulation have on the electronic structure of the surface layer, we have developed a minimal tight‐binding model for the band structure at the surface of Sr_2_RuO_4_. This model was generated by taking a tight‐binding model of the bulk of Sr_2_RuO_4_,^[^ ^18^ ^]^ and then altering the position of the d_*xy*_ vHs such that it is shifted toward the Fermi level (see Section S4, Supporting Information for details). We then include the emergent orders at a phenomenological level. The surface reconstruction and the checkerboard charge order (Figure 2) are accounted for by including a weak intraband hybridization (Δ_hyb_). The nematicity and inequivalence of the [10] and [01] lattice directions (Figure 3) are included through a nematic order parameter Δ_nem_ = δ_nem_(cos(*k*_*x*_) − cos(*k*_*y*_)), which we apply only to the d_*xy*_ orbital. A similar order parameter has previously been introduced to describe nematicity in Sr_3_Ru_2_O_7_.^[^ ^31^ ^]^ The full Hamiltonian is then

(1) $$H(k)\;\;=\;\;\left(\begin{array}{cc} H_{\text{Ru}}(k)+\Delta_{\text{nem}}{\hat{I}}_{xy} & \Delta_{\text{hyb}}\hat{I}\\ \Delta_{\text{hyb}}\hat{I} & H_{\text{Ru}}(k+Q)+\Delta_{\text{nem}}{\hat{I}}_{xy} \end{array}\right)$$

where $H_{\text{Ru}}(k)$ is the Hamiltonian for the ruthenium 4d t_2g_ bands in the unreconstructed Brillouin zone,^[^ ^18^ ^]^ and Q=(π,π) accounts for the doubling of the unit cell (for details see Section S4, Supporting Information). The inclusion of the emergent orders leads to the formation of four vHss around the M point, shown in **Figure** **4**a which shows the band structure obtained from Equation (1). We follow the notation by van Hove^[^ ^32^ ^]^ to label the four vHss: nematicity results in two saddle points, S1 and S2, on the high symmetry axis at the M point, whereas hybridization leads to a partial gap with a band maximum M and an additional saddle point around *E*_F_. The resulting complex low‐energy electronic structure around the M point is shown in a 3D representation in Figure 4b. The partial drop in the *g*(*V*) spectrum around *E*_F_, observed in Figure 2d, is naturally explained by the hybridization of the Ru d_*xy*_‐bands caused by the doubling of the unit cell. The four singularities lead to maxima in the density of states (see Figure 4c) in excellent agreement with the structure of the low‐energy *g*(*V*) spectrum. Differences, such as the magnitude of the drop, are likely a consequence of tunneling matrix element effects, which are neglected in the calculation.

### 2.4 Magnetic‐Field Tuning of the Electronic Structure

The presence of multiple vHss in the immediate vicinity of the Fermi energy offers the opportunity to drive the material through a Lifshitz transition using magnetic field and Zeeman splitting of the bands. We investigate the influence of an applied magnetic field, focusing on the behaviour of the most prominent vHs, M, at −3.4 mV. Figure 4d shows tunneling spectra recorded in magnetic fields from 0 T to 13.4 T, applied perpendicular to the surface, at *T* = 76 mK. The spectra reveal a clear splitting of the vHs with increasing magnetic field. In Figure 4e, the field‐dependence of the energy of the vHs reveals a linear behavior as expected for a Zeeman‐like splitting. We find the slope to be +0.10 mV T^−1^ for the peak moving toward the Fermi energy and −0.07 mV T^−1^ for the one moving away. The difference in slope can be attributed to an overall chemical potential shift of the d_*xy*_ band of 17 μV T^−1^. We find a *g*‐factor of g* ≈ 3 (with the splitting Δ*E* = g*μ_B_ *B*_*z*_). This value is consistent with the known Wilson ratio *W* = g*/*g* = 1.5 of bulk Sr_2_RuO_4_,^[^ ^33^, ^34^ ^]^ a surprisingly good agreement given that the Wilson ratio contains contributions from the whole Fermi surface, whereas we determine g* only for one band. From our experimental data, we can extrapolate that the vHs will cross the Fermi energy at a magnetic field of about 32 T. At this field, the surface layer is expected to undergo a Lifshitz transition. As the energy of the vHs is associated with the checkerboard charge order, once it is split in a magnetic field, the charge order becomes spin‐polarized. We show in Figure 4f that indeed also the energy at which the checkerboard charge order appears most prominently shifts with the change in the energy of the vHs, demonstrating that the two are intimately linked. Our measurements demonstrate that the electronic structure of the reconstructed surface layer of Sr_2_RuO_4_ has all the ingredients for a field‐tuned Lifshitz transition: 1) a vHs close to the Fermi energy, 2) the energy of the vHs can be tuned by magnetic field toward the Fermi energy, and 3) a Lifshitz transition of the electronic structure within reach of available magnetic fields.

## 3 Discussion

Our measurements show how the low‐energy electronic structure of a 2D layer of Sr_2_RuO_4_ is impacted by octahedral rotations and stabilizes new emergent phases close to a magnetic‐field induced Lifshitz transition. We provide a comprehensive description of the surface electronic structure through a phenomenological tight‐binding model that incorporates the two orders we detect, nematicity and checkerboard charge order and reproduces all features we observe in our data. It captures the pronounced gap‐like feature seen in tunneling spectra. The quick changes in the appearance of the nematicity in spectroscopic maps within a narrow energy interval are naturally explained by the vHss in the *k*_*x*_‐ and *k*_*y*_‐directions becoming inequivalent. While checkerboard charge order and nematicity are intimately intertwined, it is not clear which of the two dominates, if either does. Possible mechanisms include 1) nematicity driven by electronic correlations, 2) charge order driven by nesting or a lattice distortion, 3) a cooperative effect of both, and 4) an antiferromagnetic order or fluctuations which stabilize the checkerboard order and nematicity. For the first three scenarios, one would expect a significant structural distortion accompanying these, as is seen for charge density waves or nematicity in other systems. A structural distortion in the surface layer of Sr_2_RuO_4_ beyond the octahedral rotation has not been reported.^[^ ^29^ ^]^ In a scenario where magnetic order or fluctuations drive nematicity and charge order, one would still expect a structural distortion, though much smaller. Recently, evidence for nematicity in thin films of Sr_2_RuO_4_ has been reported, suggesting that it may not be limited to the surface layer.^[^ ^35^ ^]^

A comparison with Sr_3_Ru_2_O_7_ reveals intriguing parallels: Sr_3_Ru_2_O_7_ exhibits a similar (albeit larger) octahedral rotation, and the energy of the vHs as detected by ARPES is found at ≈4 meV below *E*_F_,^[^ ^36^ ^]^ close to the energy at which we find the dominant vHs in the surface layer of Sr_2_RuO_4_. Due to stronger correlations and a *g*‐factor of g* ≈ 14.6 in Sr_3_Ru_2_O_7_,^[^ ^37^ ^]^ the vHs can be tuned to the Fermi energy at 8T, whereas in the surface layer of Sr_2_RuO_4_, we find a *g*‐factor that is almost 4 times smaller. Consequently, the vHs is expected to reach the Fermi energy only at 32T. It remains to be seen whether the parallels go any further when the surface layer in Sr_2_RuO_4_ undergoes the Lifshitz transition. There are also important differences, though, including that the system we report here is strictly 2D which puts the criticality of the Lifshitz transition into a different universality class than what is expected for the bulk of Sr_3_Ru_2_O_7_. Tunneling spectra recorded on Sr_3_Ru_2_O_7_ do not reveal a clear shift of a peak as a function of magnetic field,^[^ ^28^ ^]^ possibly because quantum fluctuations play a much larger role and already have influence on the line shape of the vHs in the density of states. Previous studies of the reconstructed surface have not been able to detect signatures of superconductivity,^[^ ^21^, ^22^ ^]^ except a recent study^[^ ^38^ ^]^ where a gap‐like structure in the vicinity of the Fermi energy has been attributed to superconductivity. Tunneling spectra showing clear signatures of superconductivity with a temperature and/or magnetic field dependence consistent with bulk Sr_2_RuO_4_^[^ ^39^, ^40^, ^41^ ^]^ were either obtained from surfaces that were exposed to air leading effectively to a dirty surface much like in ARPES experiments probing the bulk electronic structure or on a different surface reconstruction than the one studied here. We do not observe any evidence for superconductivity even in tunneling spectra acquired at temperatures well below 100 mK. We have ensured that the spectroscopic resolution of our instrument is sufficient to detect superconducting gaps of materials with a similar transition temperature and gap sizes on the order of 200 μeV.^[^ ^42^, ^43^ ^]^ This indicates that the emergent electronic order suppresses superconductivity in the surface layer. This suppression may imply that the density of states of the d_*xy*_ band around *E*_F_, which becomes gapped out at the surface, and the fluctuations associated with the reconstruction play a crucial factor in superconducting pairing. This scenario naturally results in a competition of nematicity and the charge density modulations with superconductivity, reminiscent to what is found in other strongly correlated electron systems.^[^ ^5^, ^44^ ^]^ Understanding the leading instability in this highly debated material therefore undoubtedly will have to account for this susceptibility toward density wave formation as observed in other unconventional superconductors.

## 4 Conclusions

We show that the surface layer of Sr_2_RuO_4_ provides a 2D model system to study the intricate structure–property relationships of a strongly correlated electron system. We demonstrate the equivalence of checkerboard charge order and nematicity in this system, and find that the reconstructed electronic structure leads to four vHss within 5 meV of the Fermi energy. Magnetic‐field tuning of one of these vHs implies that the surface layer can be used as a well‐controlled test system to study magnetic‐field induced Lifshitz transitions, enabling detailed comparison with microscopic theories. Because the emergent surface phase is strictly 2D, limited to the surface layer, all relevant information about the electronic states is accessible spectroscopically. Magnetic‐field tuned Lifshitz transitions have been proposed to be at the heart of the quantum critical behavior in a range of heavy fermion and strongly correlated electron materials,^[^ ^45^, ^46^, ^47^ ^]^ yet the ability to spectroscopically trace the electronic structure across a field‐tuned quantum phase transition has remained elusive. Given the sensitivity of the energy of the vHs in Sr_2_RuO_4_ to uniaxial strain,^[^ ^6^ ^]^ we expect that the magnetic field at which the Lifshitz transition is extrapolated to occur here can be reduced substantially by combining uniaxial strain with magnetic field. This creates the opportunity to spectroscopically verify the role of quantum fluctuations across a magnetic field‐tuned Lifshitz transition through a detailed study of the line shape of the vHs as it is tuned across the Fermi energy. The sensitivity of the electronic structure of ruthenates to tiny structural modifications found here shows opportunities for tailoring correlated electronic phases in 2D and exploring their physics for novel electronic devices.

## Conflict of Interest

The authors declare no conflict of interest.

## Supporting information

Supporting Information

## Data Availability

The data that support the findings of this study are openly available at the St Andrews University Research Portal at https://doi.org/10.17630/fc3776f6-dcd8-4624-a497-7ef8e770bb92.
