# Supplementary material for: Magnetic‐Field Tunable Intertwined Checkerboard Charge Order and Nematicity in the Surface Layer of Sr2RuO4
Source: Adv Mater. 2021 Jun 27;33(32):2100593. doi: 10.1002/adma.202100593 (PMC11469189; doi:10.1002/adma.202100593)
Supplement: Supplementary file 1 — Supporting Information [file ADMA-33-2100593-s001.pdf]

© 2021 Wiley-VCH GmbH

ADVANCED MATERIALS

Supporting Information

for *Adv. Mater.,* DOI: 10.1002/adma.202100593

Magnetic-Field Tunable Intertwined Checkerboard Charge Order and Nematicity in the Surface Layer of Sr_2RuO_4

*Carolina A. Marques, Luke C. Rhodes, Rosalba Fittipaldi, Veronica Granata, Chi Ming Yim, Renato Buzio, Andrea Gerbi, Antonio Vecchione, Andreas W. Rost, and Peter Wahl**

Supplementary Material for

Magnetic-field tunable intertwined checkerboard charge order and nematicity in the surface layer of Sr_2RuO_4

Carolina A. Marques,[1,*] Luke C. Rhodes,[1,*] Rosalba Fittipaldi,[2]
Veronica Granata,[3] Chi Ming Yim,[1] Renato Buzio,[4] Andrea Gerbi,[4]
Antonio Vecchione,[2] Andreas W. Rost,[1,5] and Peter Wahl[1,†]

[1]School of Physics and Astronomy,
University of St Andrews, North Haugh,
St Andrews, KY16 9SS, United Kingdom
[2]CNR-SPIN, UOS Salerno, Via Giovanni Paolo II 132, Fisciano, I-84084, Italy.
[3]Dipartimento di Fisica "E. R. Caianiello",
Università di Salerno, I-84084 Fisciano, Salerno, Italy.
[4]CNR-SPIN, Corso F.M. Perrone 24, Genova, 16152, Italy
[5]Max-Planck-Institute for Solid State Research,
Heisenbergstr. 1, 70569 Stuttgart, Germany

This PDF file includes:

Materials and Methods (S1)

Bias dependence of the checkerboard charge order in topographic STM images (S2)

Differential conductance maps (S3)

Tight-binding model (S4)

Analysis of magnetic-field dependent tunneling spectra (S5)

Figs. S1 to S9

*These authors contributed equally.
†correspondence to: wahl@st-andrews.ac.uk

S1. MATERIALS AND METHODS

A. Single crystal growth.

The Sr_2RuO_4 crystals used in this work were grown by the floating-zone technique with Ru self-flux, using a commercial image furnace with double-elliptical mirrors and two 2.0kW halogen lamps (*S1*). Morphological and elemental characterization of the crystals was carried out using a Zeiss Leo EVO 50 scanning electron microscope (SEM) equipped with an Oxford INCA Energy 300 energy dispersive X-ray spectroscopy (EDS) system. The structure and crystalline quality of the samples were assessed by a high-resolution X-ray diffractometer (Panalytical, X Pert MRD), with a Cu K-α source.

B. Characterization

1. Transmission electron microscopy (TEM)

To verify the crystal quality in the surface region following cleavage of the sample in the STM, we have performed Transmission Electron Microscopy on a sample on which STM measurements had been carried out. The sample for STEM analysis was prepared by conventional gallium focused ion beam (FIB) milling using an FEI Scios focused ion beam scanning electron microscope (FIBSEM) equipped with an EDAX Hikari Super electron back-scattered diffraction (EBSD) detector. The orientation of the sample was determined by EBSD prior to milling, to cut a lamella in the [010] plane. High angle annular dark field (HAADF) images were recorded using a probe corrected FEI Themis 200 scanning/transmission electron microscope operated at 200kV.

Figure S1(a) shows a TEM image of a cross section of the sample after cleaving. It shows a uniform phase along the c-axis on the scale of $\approx$ 80nm extending up to the surface. Figure S1(b) shows a zoom in with atomic resolution. It shows the expected stacking for Sr_2RuO_4, as evidenced by the inset, where both strontium and ruthenium atoms are visible, with the Ru atoms appearing with a slightly higher intensity. Oxygen atoms are not visible. The TEM image shows no evidence of inclusions of other members of the Ruddlesden-Popper series (i.e. $Sr_{n+1}Ru_nO_{3n+1}$ with $n > 1$).

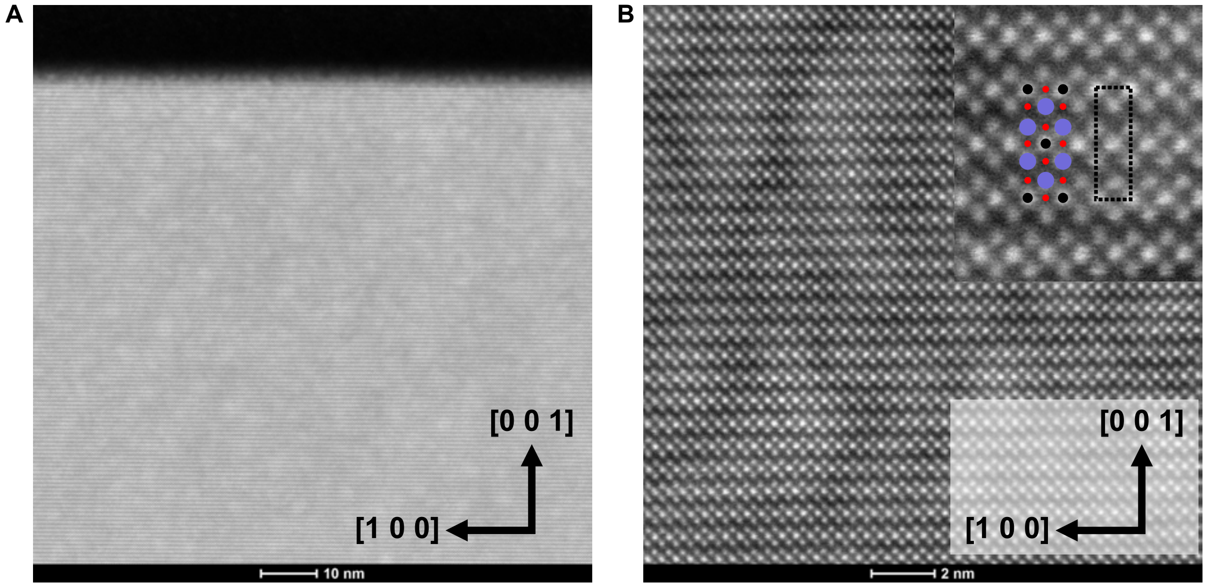

FIG. S1: TEM images along the b-axis. (a) Image of the cross-section of a sample after STM measurements have been performed on its surface, demonstrating uniformity up to the surface. (b) High-resolution image showing atomic resolution. The inset shows a zoom in of the image, with the stacking expected for Sr_2RuO_4. Superimposed to the TEM image is the atomic structure, with spheres indicating strontium (purple), ruthenium (black) and oxygen (red) atoms. The black dashed line indicates one unit cell of Sr_2RuO_4.

2. Resistance measurements

Transport measurements were performed using a four probe technique with a ^{3}He refrigerator. The resistance as a function of temperature (Fig. S2) shows a superconducting transition at $T_c = 1.5$K, in agreement with data reported in literature for good quality crystals(*S2*).

We determine a residual-resistance ratio (RRR) $\lim_{T\to 0\mathrm{K}} \frac{R(300\mathrm{K})}{R(T)} \approx 666$, extrapolated to 0K from fitting the resistance above T_c to $R(T) = R_{\mathrm{res}} + AT^2$ (Figure S2(a)). This value is comparable to that of high-purity crystals reported in the literature(*S3*).

3. Scanning tunneling microscopy (STM)

Experiments were performed in a home-built ultra-low temperature STM operating in a dilution refrigerator.(*S4*) Samples were prepared by *in-situ* cleaving at low temperatures

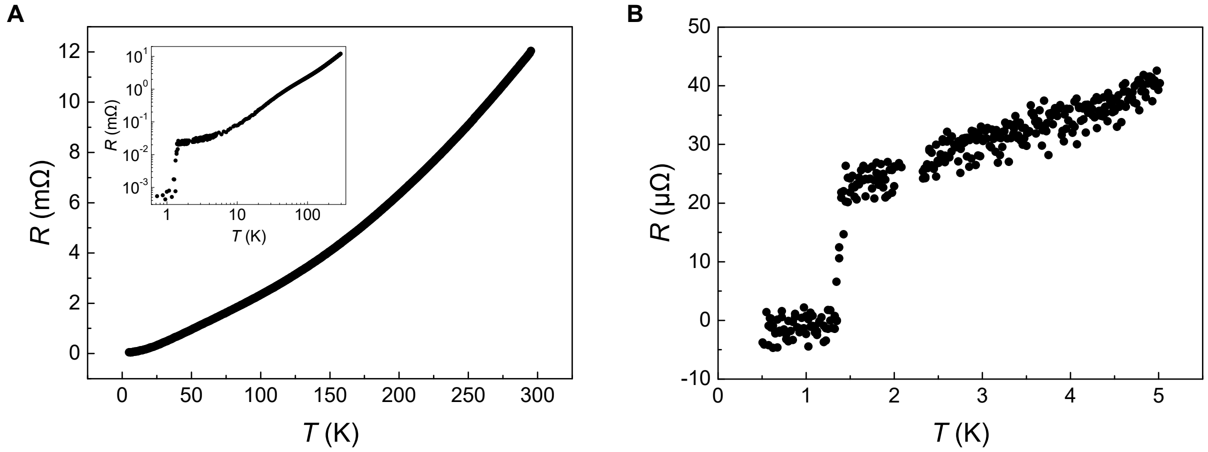

FIG. S2: **Resistance as a function of temperature of Sr_2RuO_4 with current in the a-b plane.** (a) Resistance up until room temperature. The inset shows the resistance as function of temperature in log-log scale (b) Low temperature resistance down to 0.5 K showing the superconducting transition at 1.5 K.

($\sim$ 20K) in cryogenic vacuum. We used STM tips cut from PtIr wire, and prepared them *in-situ* by field emission on a Au(111) single crystal. Bias voltages were applied to the sample, with the tip at virtual ground.

Spectroscopic measurements were performed using a lock-in amplifier to measure differential conductance $g(V)$. The bias voltage V was modulated at a frequency of $\nu = 397$Hz, the lock-in modulation V_{L}, specified as amplitude, is given in figure captions where applicable. The bias and current setpoints, V_{set} and I_{set}, are indicated for both topographies and spectroscopic maps.

After cleaving and inserting the samples into the STM head, we have always observed the reconstructed SrO termination as observed in previous reports(*S5–S8*) and as can be inferred from the appearance of the defects and the additional Fourier peaks due to the checkerboard charge order.

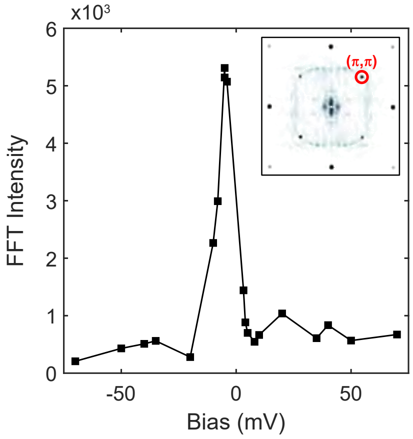

FIG. S3: **Bias dependence of the checkerboard charge order in topographic STM images.** Intensity of the peak in the Fourier transformation $\tilde{z}(\mathbf{q})$ of topographic images $z(\mathbf{r})$ corresponding to the checkerboard charge order at $\mathbf{q}_{\mathrm{ckb}} = (\pi, \pi)$ for bias voltages between $+/-70$mV. The inset shows a typical Fourier transformation taken with -5mV, where the peak at $\mathbf{q}_{\mathrm{ckb}}$ is indicated by a red circle. This data was taken at 2K and the tunneling resistance was kept at 100MΩ for all measurements.

S2. BIAS DEPENDENCE OF THE CHECKERBOARD CHARGE ORDER IN TOPOGRAPHIC STM IMAGES

The appearance of the checkerboard modulation in topographies is bias dependent, as seen in Figures 2(a) and (b) of the main manuscript. Here we show the bias dependence of the appearance of the checkerboard charge order in the voltage range between -70mV and $+70$mV. We plot the intensity $\tilde{z}(\mathbf{q}_{\mathrm{ckb}})$ of the peak in the Fourier transformation associated with the checkerboard order ($\mathbf{q}_{\mathrm{ckb}} = (\pi, \pi)$) as a function of applied bias voltage V in Figure S3. The intensity of the checkerboard charge order has a sharp maximum around -5mV, with its intensity decreasing by a factor of 25 at -70mV and of 8 at $+70$mV. Using this information, for spectroscopic maps shown here, we have chosen a tunneling setpoint at small positive bias voltages where the checkerboard charge order is weak to minimize the setpoint effect.

S3. DIFFERENTIAL CONDUCTANCE MAPS

In this section, we show the real space conductance maps underpinning the data shown in Fig. 3(d) and how it has been processed.

A. Real space maps

The topographic image acquired simultaneously with the map taken at 2K is shown in Figure S4(a) and the corresponding real space differential conductance map, $g(\mathbf{r}, V) = \mathrm{d}I/\mathrm{d}V(\mathbf{r}, V)$, for $V = -3.4$mV in Figure S4(b). The checkerboard charge order is clearly visible. In addition quasiparticle interference (QPI) effects are observed around the defect seen in the topography.

B. Processing of differential conductance maps

In Figure S4 we show images following each step of the data processing. The raw Fourier transformation of the $g(\mathbf{r}, V)$ map is displayed in Fig. S4(c). It shows clear signatures of the atomic peaks at $(\pm 2\pi, 0)$ and $(0, \pm 2\pi)$ (in units of $1/a$, where a is the lattice constant of the bulk tetragonal unit cell), as well as of the checkerboard charge order at $(\pm\pi, \pm\pi)$ and $(\pm\pi, \mp\pi)$. There are in addition weak higher order peaks. The Fourier transformation is first corrected for any linear drift by mapping the atomic peaks by a linear transformation onto a perfect square (Fig. S4(d)). Next, the resulting images are mirror-symmetrized along the horizontal or vertical direction (Fig. S4(e)). Finally, to suppress the high intensity at the centre of the image due to the distribution of defects, the image is multiplied by 1 minus a two-dimensional Gaussian function with a standard deviation of 4 pixels (Fig. S4(f)).

This procedure was implemented for all layers of the map. Fig. S5 shows the Fourier transformation after processing of the same $g(\mathbf{r}, V)$ map at different energies, between -8.2mV and $+7.8$mV. The intensity of the $(\pm\pi, \pm\pi)$ peaks is strongly energy dependent, showing maximum intensity at -3.4mV (highlighted by a red box). Additionally, we observe an anisotropy in the intensities of the $(2\pi$,0) and (0,$2\pi)$ peaks, as is indicated in Fig. S5 by the circles drawn with solid red and dashed white lines. It can be seen that the high intensity switches from one direction to the other with energy. This reflects the atomic scale symmetry

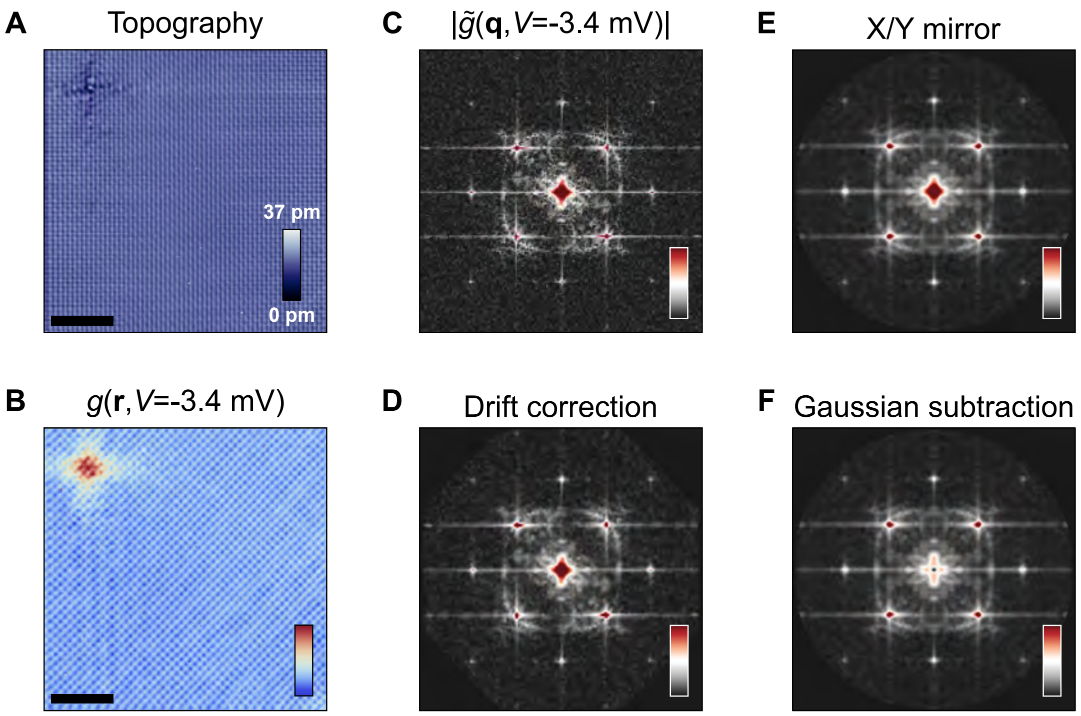

FIG. S4: **Processing of differential conductance maps.** (a) Topography acquired simultaneously with map, showing one defect in the top left corner. Scale bar: 5nm (V_{set} = 7.8mV, I_{set} = 225pA). (b) Real space $g(\mathbf{r}, V)$ at $V = -3.4$ mV. (c) Absolute value of its Fourier transformation, $|\tilde{g}(\mathbf{q}, V)|$, (d) after drift correction, (e) after symmetrizing along the horizontal (or vertical) direction, and (f) after subtraction of a gaussian function with a width of 4 pixels at the centre of the image ($V_{\text{L}} = 800\mu$V, $T = 2$K, $B_z = 0$T).

breaking shown in Fig. 4(b) and (c) in the main text and is linked to the nematicity.

C. Phase-referenced Fourier transformation

In figs. 3(d) and 6(c) of the main text, we show phase-referenced Fourier transformations to deduce the characteristic energy scale of the checkerboard charge order and the relative phase at positive and negative bias voltages. The Fourier transformation $\tilde{g}(\mathbf{q}, V)$ of a differential conductance map $\tilde{g}(\mathbf{r}, V)$ which shows the atomic lattice exhibits two pairs of Bragg peaks at $\mathbf{q}_{\text{at}} = (\pm 2\pi, 0)$ and $(0, \pm 2\pi)$, indicated by a black circle in Figure S6(a). The checkerboard order shown in Figure 3(b), (c) of the main text has a wavelength that is

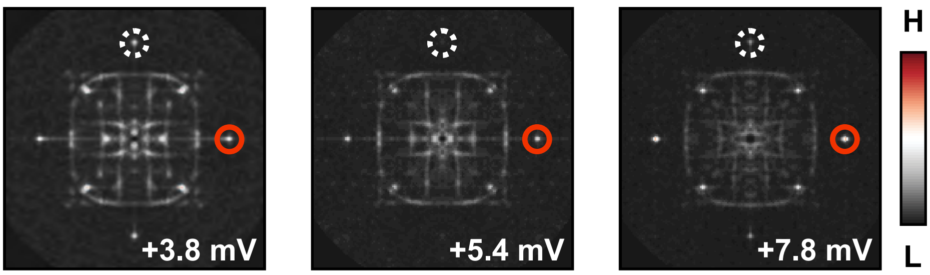

FIG. S5: **Energy layers from the $\tilde{g}(\mathbf{q}, V)$ map** shown in Fig. 3(d) of the main text and Fig. S4 for the data at 2K. The intensity of the peak at $\mathbf{q}_{\text{CKB}} = (\pi, \pi)$ due to the checkerboard charge order is strongly energy dependent. The layer corresponding to the energy of the vHs is highlighted by a red box, where the peaks at $\mathbf{q}_{\text{CKB}}$ exhibit the highest intensity. Additionally, the intensity of the Bragg peaks $\mathbf{q}_{\mathbf{at}} = (0, \pm 2\pi)$ and $(\pm 2\pi, 0)$ switches with energy due to the nematicity (cf. fig. 4(b), (c) of the main text). For each layer, the higher intensity peak is highlighted by a red circle, and the lower intensity peak with a dashed white circle. The range of the color bar is the same for all images.

$\sqrt{2}$ times larger, with the unit vector 45° rotated. The periodicity due to the checkerboard charge order shows up as four peaks at $\mathbf{q}_{CKB} = (\pm\pi, \pm\pi)$ and $\mathbf{q}_{CKB} = (\pm\pi, \mp\pi)$, one of which is indicated by the red circle in Fig. S6(a). The amplitude of this checkerboard order is reflected in the intensity of the Fourier peak, $|\tilde{g}(\mathbf{q}_{CKB}, V)|$, as shown in Fig. S5. We can determine the characteristic energy scale of the checkerboard modulation as a function of energy by plotting, $|\tilde{g}(\mathbf{q}_{CKB}, V)|$ as a function of applied bias V, however losing the phase information $\phi(\mathbf{q}, V)$ contained in the Fourier transformation,

$$\tilde{g}(\mathbf{q}, V) = \sqrt{\frac{\Delta x \Delta y}{N_x N_y}} \mid \tilde{g}(\mathbf{q}, V) \mid e^{i\phi(\mathbf{q}, V)}, \tag{S1}$$

where Δx and Δy are the size of the map in along the x and y directions and N_x and N_y are the number of pixels in each direction. While analyzing the phase $\phi(\mathbf{q}, V)$ itself is possible, it suffers from an arbitrary global phase factor. To remove this global phase factor, we use a phase-referenced Fourier transformation (PR-FT)(*S9*)

$$\tilde{g}^{\mathrm{R}}(\mathbf{q}, V) = \frac{\tilde{g}(\mathbf{q}, V)}{e^{i\phi(\mathbf{q}, V_0)}} = \sqrt{\frac{\Delta x \Delta y}{N_x N_y}} \mid \tilde{g}(\mathbf{q}, V) \mid e^{i(\phi(\mathbf{q}, V) - \phi(\mathbf{q}, V_0))}. \tag{S2}$$

In this PR-FT, the phase at each $\mathbf{q}$-vector is referenced to the phase at a specific energy V_0, removing the global phase factor. This allows tracking of the change in phase as a function of energy relative to that layer by simply plotting the real part of the PR-FT,

$$Re\left[\tilde{g}^{\mathrm{R}}(\mathbf{q}, V)\right] = \sqrt{\frac{\Delta x \Delta y}{N_x N_y}} \mid \tilde{g}(\mathbf{q}, V) \mid \cos\left(\phi(\mathbf{q}, V) - \phi(\mathbf{q}, V_0)\right). \tag{S3}$$

Here, we reference the phase to the map layer at $V_0 = -3.4$mV. The real part of the PR-FT images, Figure S6(c), $\mathrm{Re}[\tilde{g}^{\mathrm{R}}((\pi, \pi), V)]$ allows us to determine if there is a phase shift between the checkerboard order at different energies as well as the relative amplitude. A phase reversal means a change in sign. At the reference energy, V_0, $\mathrm{Re}[\tilde{g}^{\mathrm{R}}((\pi, \pi), V_0]$ will be positive by definition. Fig. S6(c) shows $\mathrm{Re}[\tilde{g}^{\mathrm{R}}((\pi, \pi), V = +3\mathrm{mV}]$, where the peaks at $\mathbf{q}_{\mathrm{CKB}}$ appear with a negative sign, evidencing a phase shift with respect to the charge modulation at $V_0 = -3.4$mV. Figure 3(d) of the main text shows that the checkerboard order appears predominantly at $V = -3.5$mV and $V = 3.5$mV with opposite phase.

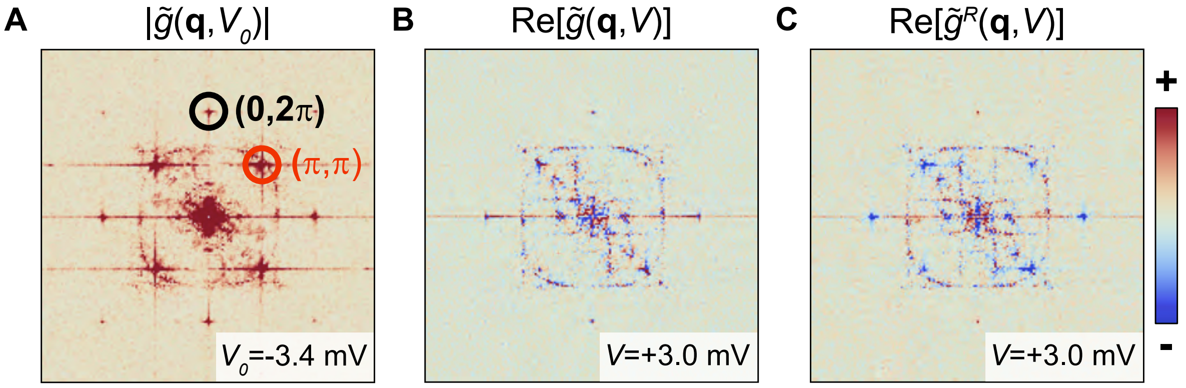

FIG. S6: **Phase-referenced Fourier transformation.** (a) Amplitude $|\tilde{g}(\mathbf{q}, V_0 = -3.4\text{mV})|$ of the Fourier transformation. (b) Real part $\mathrm{Re}[\tilde{g}(\mathbf{q}, V = +3.0\text{mV})]$, it can be seen that the peaks at (π, π) and $(-\pi, \pi)$ have opposite sign. (c) Real part of the phase-referenced Fourier transformation (PR-FT), $\mathrm{Re}[\tilde{g}^{\mathrm{R}}(\mathbf{q}, V = +3.0\text{mV})]$, showing the reversed sign of the peaks at (π, π) and $(-\pi, \pi)$ compared to panel A (map parameters as in fig. S4).

S4. TIGHT-BINDING MODEL

The tight-binding model for the surface of Sr_2RuO_4 may be defined, in an analogous manner to that of the bulk, via hopping associated with the three bands derived from the ruthenium t_{2g} orbitals (*S10*),

$$H^{\sigma}(\mathbf{k}) = \begin{pmatrix} E_{xz}(\mathbf{k}) & \gamma(\mathbf{k}) - \sigma i\eta & i\eta \\ \gamma(\mathbf{k}) + \sigma i\eta & E_{yz}(\mathbf{k}) & -\sigma\eta \\ -i\eta & -\sigma\eta & E_{xy}(\mathbf{k}) \end{pmatrix}. \tag{S4}$$

This Hamiltonian includes nearest neighbour hoppings between the d_{xz} and d_{yz} orbitals as well as up to third nearest neighbour hoppings between d_{xy} orbitals,

$$E_{xz}(\mathbf{k}) = -2t_1\cos(k_x) - 2t_2\cos(k_y) - \mu,$$
$$E_{yz}(\mathbf{k}) = -2t_2\cos(k_x) - 2t_1\cos(k_y) - \mu,$$
$$E_{xy}(\mathbf{k}) = -2t_3(\cos(k_x) + \cos(k_y)) - 4t_4\cos(k_x)\cos(k_y) - 2t_5(\cos(2k_x) + \cos(2k_y)) - \mu_c. \tag{S5}$$

The off diagonal term, $\gamma(\mathbf{k})$, describes inter-orbital hopping between the degenerate d_{xz} and d_{yz} states and is written as $\gamma(\mathbf{k}) = -4t_{\text{inter}}\sin(k_x)\sin(k_y)$. The rest of the off-diagonal terms arise from the spin-orbit interaction, where η is the spin-orbit coupling constant and σ is defined as $+1$ for up spins ($\uparrow$) and -1 for down spins ($\downarrow$).

Using this basis, a tight binding model for the electronic structure can be defined via the Hamiltonian

$$H_{\text{Ru}}(\mathbf{k}) = \begin{pmatrix} H^{\uparrow}(\mathbf{k}) & 0 \\ 0 & H^{\downarrow}(\mathbf{k}) \end{pmatrix}, \tag{S6}$$

representing now a 6×6 matrix. For the surface, we must account for the doubling of the unit cell due to the additional octahedral rotation. This surface tight binding model is then given by the 12×12 matrix

$$H_{\text{surf}}(\mathbf{k}) = \begin{pmatrix} H_{\text{Ru}}(\mathbf{k}) & 0 \\ 0 & H_{\text{Ru}}(\mathbf{k}+\mathbf{Q}) \end{pmatrix}, \tag{S7}$$

with $\mathbf{Q} = (\pi, \pi)$.

For the bulk Fermi surface, presented in Fig. 1(b) of the main text, we calculate the Eigenvalues using Eq. S6 with the hopping parameters from Ref. *S10.*

$$t_1 = 0.15\text{eV} \qquad t_2 = 0.1t_1 \qquad t_3 = 0.8t_1 \qquad t_4 = 0.3t_1 \qquad t_5 = 0\text{eV}$$
$$t_{\text{inter}} = 0.01t_1 \qquad \mu = 1.0t_1 \qquad \mu_c = 1.1t_1 \qquad \eta = 0.1t_1.$$

Here t_1 is set to 150 meV and all other parameters are defined relative to t_1. For the Fermi surface presented in Fig. 1(d) we change $t_5 = 0.1t_1$, $\mu_c = 0.75t_1$ and $\mu = 0.82t_1$ in order to describe a system with a vHs located just below the Fermi level.

For the tight-binding description of the surface electronic structure, presented in Fig. 5, we use the hopping parameters

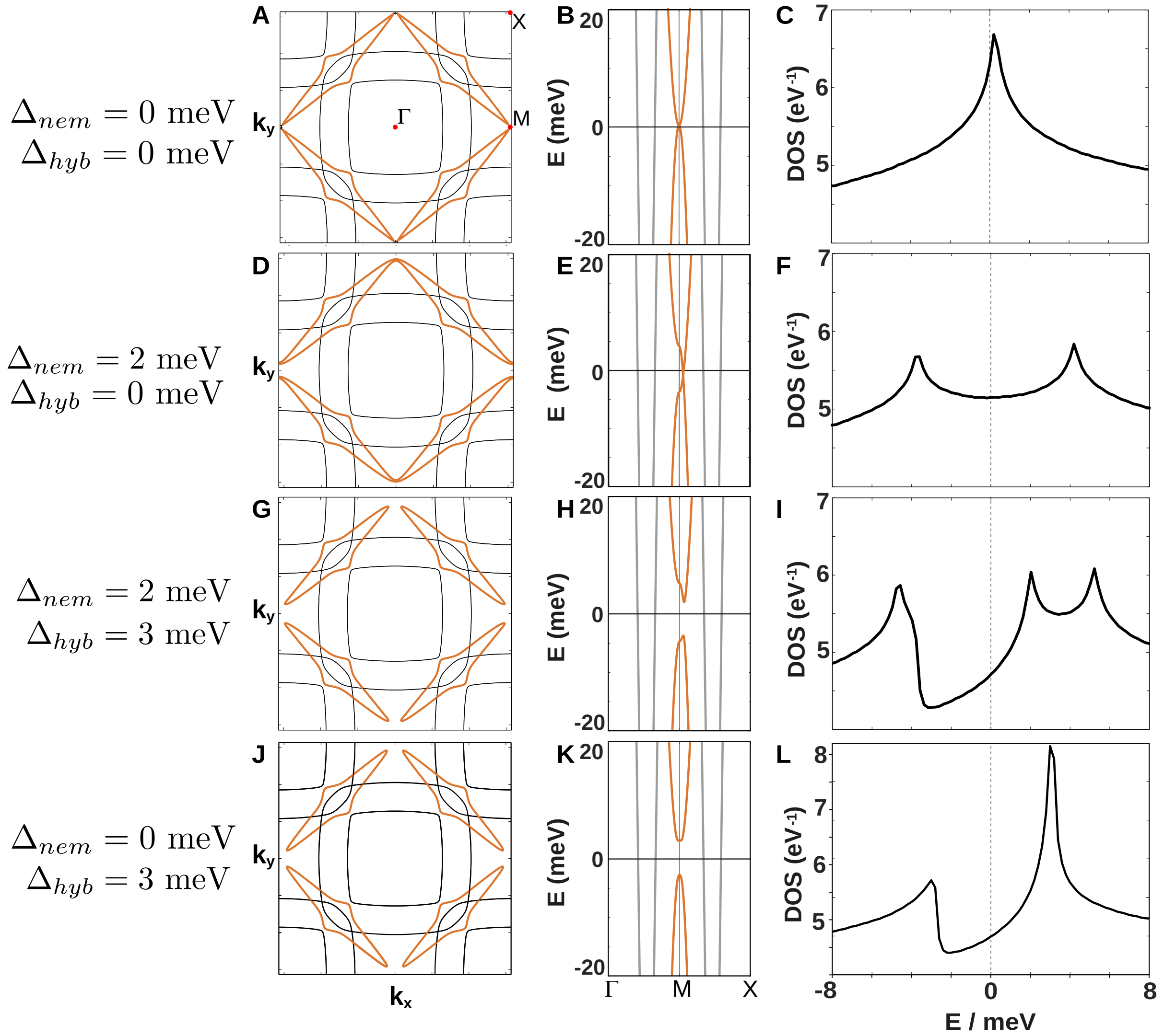

FIG. S7: Tight-binding model of the surface of Sr_2RuO_4. (a)-(c) Fermi surface, band dispersion along Γ-M-X and density of states between ±8meV for a model without nematicity ($\Delta_{\rm nem} = 0$meV and hybridization of bands due to the reconstruction $\Delta_{\rm hyb} = 0$meV. (d)-(f) Equivalent plots with nematicity included, $\Delta_{\rm nem} = 2$meV, but no hybridization between the bands due to the reconstruction $\Delta_{\rm hyb} = 0$meV. (g)-(i) Equivalent plots with nematicity $\Delta_{\rm nem} = 2$meV (as before) and a non-zero hybridization $\Delta_{\rm hyb} = 3$meV. (j)-(l) Equivalent plots with finite $\Delta_{\rm hyb}$ only.

$$t_1 = 0.15\text{eV} \qquad t_2 = 0.1t_1 \qquad t_3 = 0.8t_1 \qquad t_4 = 0.3t_1 \qquad t_5 = 0.095t_1$$
$$t_{\rm inter} = 0.01t_1 \qquad \mu = 0.75t_1 \qquad \mu_c = 0.812t_1 \qquad \eta = 0.1t_1.$$

This places the vHs just above the Fermi level and slightly decreases the Fermi wave

vector, k_F, of the d_{xz} and d_{yz} bands as suggested by ARPES measurements (*S11*). We note that the shape of the d_{xy} related pockets observed in both ARPES and DFT are slightly larger than in our model, however this difference does not affect the conclusions drawn here. We then introduce a hybridisation between the two Ru sites, $\Delta_{\text{hyb}} = 3\text{meV}$, as off-diagonal elements in Eq. S7,

$$H_{\text{surf}}(\mathbf{k}) = \begin{pmatrix} H_{\text{Ru}}(\mathbf{k}) & \Delta_{\text{hyb}}\hat{I} \\ \Delta^*_{\text{hyb}}\hat{I} & H_{\text{Ru}}(\mathbf{k}+\mathbf{Q}) \end{pmatrix}, \tag{S8}$$

and include a phenomenological C_4 symmetry breaking term $\Delta_{\text{nem}}(\mathbf{k}) = \delta_{\text{nem}}(\cos(k_x) - \cos(k_y))$ specifically to the d_{xy} orbital in H_{Ru}, with $\delta_{\text{nem}} = 2\text{meV}$, to produce the full Hamiltonian defined in Eq. 1 of the main text. A similar nematic term has been discussed for $Sr_3Ru_2O_7$ previously.(*S12*)

$$H_{\text{surf}}(\mathbf{k}) = \begin{pmatrix} H_{\text{Ru}}(\mathbf{k}) + \Delta_{\text{nem}}(\mathbf{k})\hat{I}_{xy} & \Delta_{\text{hyb}}\hat{I} \\ \Delta^*_{\text{hyb}}\hat{I} & H_{\text{Ru}}(\mathbf{k}+\mathbf{Q}) + \Delta_{\text{nem}}(\mathbf{k}+\mathbf{Q})\hat{I}_{xy} \end{pmatrix}. \tag{S9}$$

The density of states presented in Fig. 5(c) has been calculated via

$$N_0(\omega) = -\frac{1}{\pi}\text{Tr}\big[\text{Im}\big[\sum_{\mathbf{k}} G(\mathbf{k},\omega)\big]\big]. \tag{S10}$$

Here, $G(\mathbf{k},\omega)$ is the Green's function defined as $G(\mathbf{k},\omega) = \frac{1}{\omega - H(\mathbf{k}) + i\Gamma}$, where ω is the energy and Γ a broadening parameter. We use a $\mathbf{k}$-grid of 4096x4096 lattice points and $\Gamma = 0.1\text{meV}$.

Fig. S7 shows the Fermi surface, band structure and DOS given by the tight-binding model described above. Fig. S7(a)-(c) show the case where only the doubling of the unit cell is taken into account (Eq. S7). The vHs was put above, but very close to, E_{F} and it appears as a sharp peak with logarithmic divergence, cut off by the broadening parameter Γ. Fig. S7(d)-(f) show the case were only the C_4-symmetry breaking term is included. The d_{xy} band becomes C_2-symmetric, and the vHs splits into two peaks, one above E_{F} and another below, although no gap opens around the Fermi energy. Fig. S7(g)-(i) show the case of eq. S9, where both the nematic term and the hybridization potential are included. A gap is opened between the d_{xy} bands, creating four vHs. The calculated DOS reproduces the measured differential conductance spectrum, as shown in Fig. 5 of the main text.

In Fig. S8, we present the DOS calculated using the surface tight-binding model in the presence of a magnetic field. To simulate this, we introduce a Zeeman splitting term to the

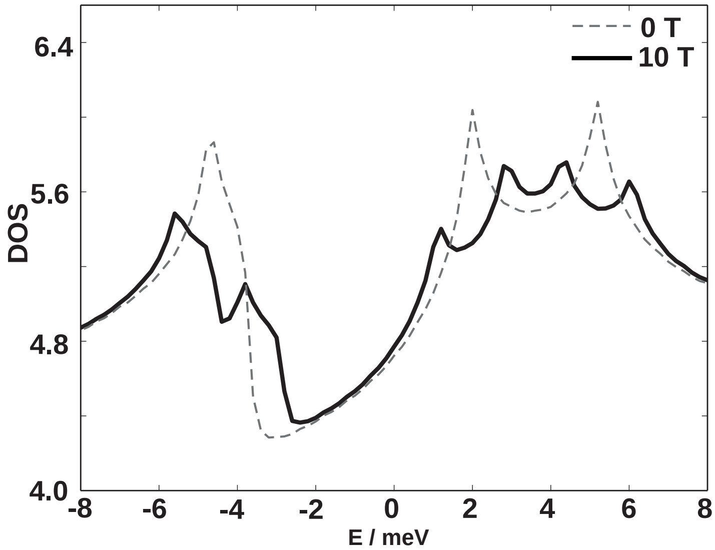

FIG. S8: DOS calculated using the surface tight binding model with a Zeeman splitting term due to a magnetic field $B_z = 10\text{T}$. The DOS calculated with $B_z = 0\text{T}$ is also shown for comparison.

Hamiltonian

$$H_{\text{Field}}(\mathbf{k}) = H_{\text{surf}}(\mathbf{k}) + \frac{\sigma}{2} g^* \mu_{\text{B}} B_z \hat{I}. \tag{S11}$$

Here $\sigma = +1$ for up spin states and -1 for down spin states. g^* has been set to 3, as determined experimentally and discussed in the main text, $\mu_{\text{B}} = 5.788 \cdot 10^{-5}$ eVT^{-1} and B_z is the magnetic field strength in the z-direction. The introduction of a magnetic field reduces the peak height of the vHs's.

S5. ANALYSIS OF MAGNETIC-FIELD DEPENDENT TUNNELING SPECTRA

Each $g(\mathbf{r}, V)$ spectrum shown in Figure 6(a) of the main text is obtained from an average of 10 spectra. All spectra were acquired with the same setpoint conditions before turning off the feedback loop. To determine the energy of the van Hove singularity from the peak positions in the spectra, we first subtract a background from the spectra, and then fit the positions of the dominant peaks. To describe the background, we fit an arc tangent and a constant ($f(V) = a \cdot \arctan[(V - V_0)/\Gamma] + c$) to the background in the data at 13.4T to describe the gap edge at negative energies and subtract the resulting function $f(V)$ as a base

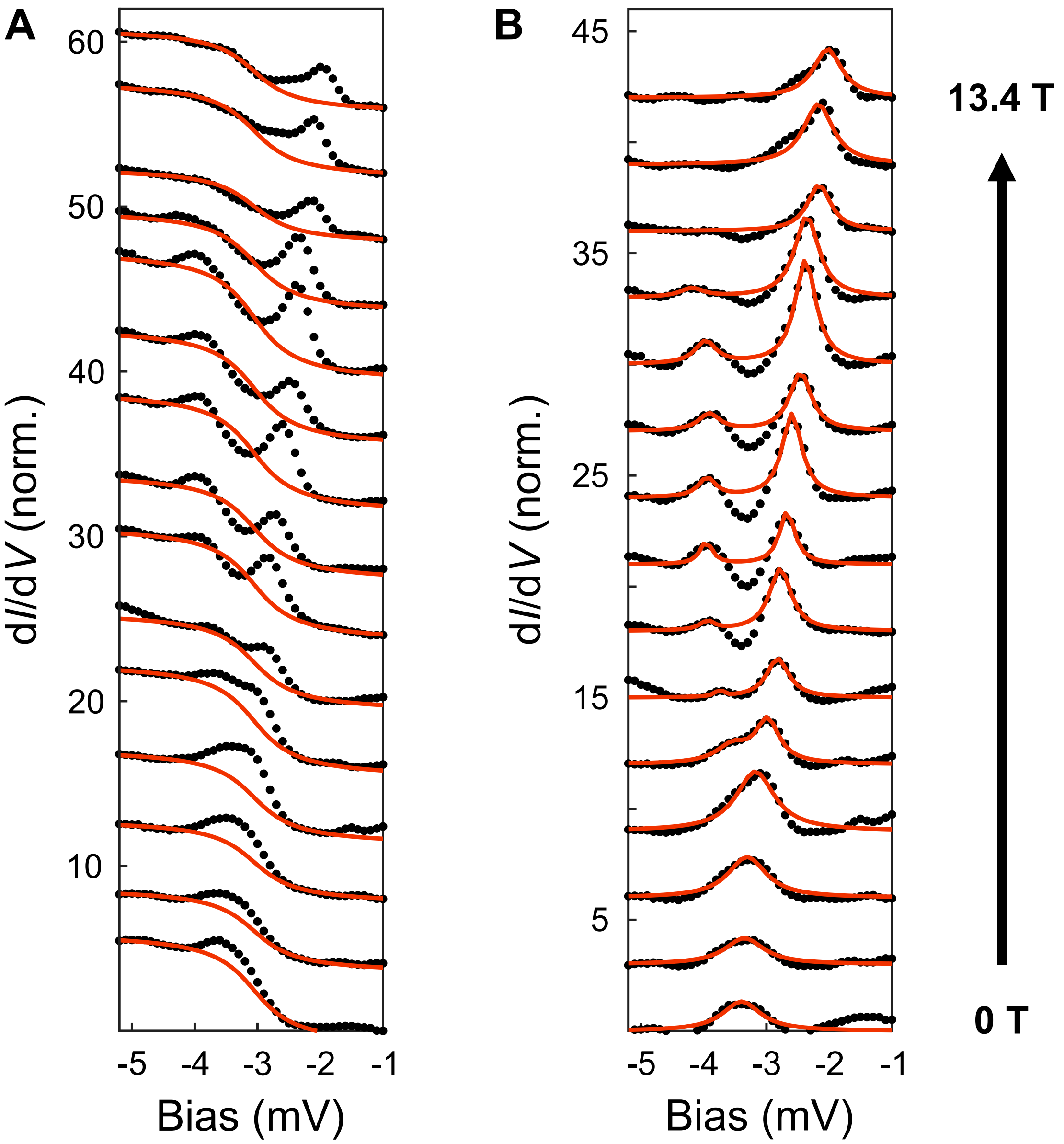

FIG. S9: **Determination of splitting of the vHs in magnetic field.** (a) Differential conductance spectra $g(\mathbf{r}, V)$ in magnetic fields of $B = 0 \ldots 13.4$T, with the background fit shown as red lines. (b) $g(\mathbf{r}, V)$ spectra after subtraction of the background. The red lines show the lorentzian fits to extract the peaks positions.

line. For the data at other fields, we fit the values of a and c to the background using the same arctangent function, but keep V_0 and Γ fixed at the values of the fit at 13.4T, Figure S9(a). To determine the energy of the van Hove singularity, we fit a Lorentzian to the peak in the spectrum, Figure S9(b).

S1. R. Fittipaldi, A. Vecchione, S. Fusanobori, K. Takizawa, H. Yaguchi, J. Hooper, R. Perry, and Y. Maeno, Journal of Crystal Growth **282**, 152 (2005), ISSN 0022-0248, URL http://www.sciencedirect.com/science/article/pii/S0022024805005841.

S2. M. E. Barber, A. S. Gibbs, Y. Maeno, A. P. Mackenzie, and C. W. Hicks, Phys. Rev. Lett. **120**, 076602 (2018), URL https://link.aps.org/doi/10.1103/PhysRevLett.120.076602.

S3. A. P. Mackenzie, R. K. W. Haselwimmer, A. W. Tyler, G. G. Lonzarich, Y. Mori, S. Nishizaki, and Y. Maeno, Phys. Rev. Lett. **80**, 161 (1998), URL https://link.aps.org/doi/10.1103/PhysRevLett.80.161.

S4. U. R. Singh, M. Enayat, S. C. White, and P. Wahl, Review of Scientific Instruments **84**, 013708 (2013).

S5. B. Barker, S. Dutta, C. Lupien, P. McEuen, N. Kikugawa, Y. Maeno, and J. Davis, Physica B: Condensed Matter **329-333**, 1334 (2003), ISSN 09214526, URL http://linkinghub.elsevier.com/retrieve/pii/S0921452602021580.

S6. Y. Pennec, N. J. C. Ingle, I. S. Elfimov, E. Varene, Y. Maeno, A. Damascelli, and J. V. Barth, Phys. Rev. Lett. **101**, 216103 (2008), ISSN 0031-9007, 1079-7114, URL https://link.aps.org/doi/10.1103/PhysRevLett.101.216103.

S7. B. Stöger, M. Hieckel, F. Mittendorfer, Z. Wang, M. Schmid, G. S. Parkinson, D. Fobes, J. Peng, J. E. Ortmann, A. Limbeck, et al., Phys. Rev. B **90**, 165438 (2014), ISSN 1098-0121, 1550-235X, URL https://link.aps.org/doi/10.1103/PhysRevB.90.165438.

S8. Z. Wang, D. Walkup, P. Derry, T. Scaffidi, M. Rak, S. Vig, A. Kogar, I. Zeljkovic, A. Husain, L. H. Santos, et al., Nature Physics **13**, 799 (2017), ISSN 1745-2473, 1745-2481, URL http://www.nature.com/articles/nphys4107.

S9. S. Chi, W. N. Hardy, R. Liang, P. Dosanjh, P. Wahl, S. A. Burke, and D. A. Bonn, arXiv:1710.09088 [cond-mat] (2017), arXiv: 1710.09088, URL http://arxiv.org/abs/1710.09088.

S10. T. Scaffidi, J. C. Romers, and S. H. Simon, Phys. Rev. B **89**, 220510 (2014), URL https://link.aps.org/doi/10.1103/PhysRevB.89.220510.

S11. A. Tamai, M. Zingl, E. Rozbicki, E. Cappelli, S. Riccò, A. de la Torre, S. McKeown Walker, F. Y. Bruno, P. D. C. King, W. Meevasana, et al., Phys. Rev. X **9**, 021048 (2019), URL

https://link.aps.org/doi/10.1103/PhysRevX.9.021048.

S12. C. Puetter, H. Doh, and H.-Y. Kee, Phys. Rev. B **76**, 235112 (2007), ISSN 1098-0121, 1550-235X, URL https://link.aps.org/doi/10.1103/PhysRevB.76.235112.
